# SG-APSIC1053: Detection of SARS-COV-2 in nasopharyngeal swags with MALDI-TOF MS and machine learning

**DOI:** 10.1017/ash.2023.9

**Published:** 2023-03-16

**Authors:** Irina Kadyrova, Svetlana Kolesnichenko, Ilya Korshukov, Yevgeniya Kolesnikova, Valentina Barkhanskaya, Alyona Lavrinenko, Aidana Sultanbekova, Sergey Yegorov, Dmitriy Babenko

**Affiliations:** Karaganda Medical University, Kazakhstan; Karaganda, Karaganda Medical University, Kazakhstan; Karaganda, Karaganda Medical University, Kazakhstan; Karaganda, Karaganda Medical University, Kazakhstan; Karaganda, Karaganda Medical University, Kazakhstan; Karaganda, Karaganda Medical University, Kazakhstan; Karaganda, Karaganda Medical University, Kazakhstan; Hamilton, McMaster University, Canada; Karaganda, Karaganda Medical University, Kazakhstan

## Abstract

**Objectives:** The widespread distribution of SARS-CoV-2 and its high contagiousness pose a challenge for researchers seeking to develop a rapid and cost-effective screening method to identify carriers of this virus. RT-PCR is considered the gold standard for detecting viral RNA in nasopharyngeal swabs, but it is time-consuming and requires constant changes in the primer composition due to the mutation of SARS-CoV-2 strains. We propose a method for the detection of SARS-CoV-2 in nasopharyngeal swabs using MALDI-TOF MS and machine learning. **Methods:** Nasopharyngeal swabs from patients with PCR-confirmed COVID-19 and control participants were tested (130 and 80 swabs, respectively) with MALDI-TOF MS MicroFlex LT using the HCCA matrix. MALDI spectra were preprocessed in R version 4.1.2 software with the MALDIquant R package using the workflow: sqrt transformation, wavelet smoothing, SNIP-based base removal, and PQN intensity calibration. Peaks were detected with MAD algorithms with following Peak alignment on the following parameters: minFreq 70% and tolerance 0.005. Machine learning was performed with the rtemis r package on GLM, random forest, and XGBoost models. **Results:** These models were characterized by specificity, sensitivity, and F1 score. GLM models (specificity 1 and sensitivity 0.5) showed a low F1 score of 0.71. However, the random forest and XGBoost models demonstrated sensitivity, specificity, and F1 score equaling 1. **Conclusions:** We propose a screening method for SARS-CoV-2 detection (sensitivity 1 and specificity 1). This methodology combines the analysis of nasopharyngeal swab samples using MALDI-TOF-MS with machine learning. It is suitable for screening patients with COVID-19 at the first stages of diagnosis. Random forest and XGBoost models demonstrated sensitivity, specificity, and F1 scores equaling 1.

